# A Retrospective Evaluation of the Effectiveness of Thoracic Sympathetic Block: A Three-Year Experience

**DOI:** 10.3390/jcm15103771

**Published:** 2026-05-14

**Authors:** Dostali Aliyev, İbrahim Aşık

**Affiliations:** 1 Department of Anesthesiology and Reanimation, Division of Algology, Faculty of Medicine, Tobb Etü Hospital, 06510 Ankara, Turkey; 2 Department of Anesthesiology and Reanimation, Division of Algology, Faculty of Medicine, Ankara University, 06560 Ankara, Turkey

**Keywords:** sympathetic nerve block, radiofrequency ablation, CRPS (complex regional pain syndromes)

## Abstract

Background: Thoracic sympathetic block (TSB) is an interventional pain management technique commonly used in the treatment of chronic pain conditions associated with sympathetic nervous system dysfunction. Its effectiveness in patients who are refractory to conservative therapies remains clinically important. This study aims to retrospectively evaluate the effectiveness and safety of fluoroscopy-guided thoracic sympathetic block in patients with chronic pain who did not achieve adequate relief with conservative treatment modalities. This study provides real-world 12-month follow-up data using both the Numeric Rating Scale and the McGill Pain Questionnaire to enable a multidimensional assessment of long-term treatment outcomes. Study Design: Retrospective. Methods: A total of 22 patients who presented to the Algology Polyclinic at Ankara University’s İbn-i Sina Hospital between 2017 and 2021, had undergone thoracic sympathetic block, had a NRS score of ≥7, and had completed the McGill Pain Questionnaire, were included in this study. Interventional procedures were performed under fluoroscopic guidance. NRS and MPQ scores were recorded before the procedure and at the 1st, 6th, and 12th months post-intervention. Statistical analysis was performed using the Paired Samples *t*-test. The primary outcome was the change in NRS score from baseline to 12 months after the procedure. Secondary outcomes included changes in NRS and MPQ scores. Results: Of the participants, 54.5% were diagnosed with CRPS, 27.3% with postherpetic neuralgia, and 18.2% with malignancy. The mean NRS score decreased significantly from 8.59 pre-intervention to 2.05 in the 12th month after the procedure (*p* < 0.001). Similarly, MPQ scores showed a significant reduction. No procedure-related complications were observed. Conclusions: Fluoroscopy-guided thoracic sympathetic block is a safe and effective interventional option for long-term pain control in patients with chronic pain syndromes, particularly those with complex regional pain syndrome, postherpetic neuralgia, and malignancy-related pain.

## 1. Introduction

Sympathetic nerve blocks are widely utilized in the management of chronic pain. In clinical practice, patients who benefit from a diagnostic block, typically performed using a local anesthetic, may generally undergo procedures involving neurolytic agents or radiofrequency ablation, depending on the nature of the pain and underlying condition (e.g., cancer-related pain). Indications for thoracic sympathetic block include complex regional pain syndromes (CRPS), post-amputation pain syndromes, neuropathic pain syndromes, and postherpetic neuralgia. Thoracic sympathetic block is typically performed under fluoroscopic or computed tomography guidance by targeting the thoracic sympathetic chain at the upper thoracic vertebral levels. The procedure is generally indicated in patients with sympathetically maintained pain and may be followed by neurolytic block or radiofrequency ablation when a diagnostic block provides substantial pain relief. Contraindications include local infection at the injection site, coagulopathy, and inability to cooperate during the procedure. Potential complications include pneumothorax, vascular puncture, local anesthetic toxicity, and transient Horner syndrome, although these events are uncommon when the procedure is performed under image guidance.

The present study aims to retrospectively evaluate the effectiveness of thoracic sympathetic block performed under fluoroscopic guidance in patients with persistent pain due to the aforementioned conditions who could not achieve adequate outcomes from conservative treatment approaches.

## 2. Materials and Methods

Ethical approval for this study was granted by the Ankara University Faculty of Medicine Human Research Ethics Committee (Decision No: İ5-343-21, 16 June 2021). Written informed consent is routinely obtained from all patients prior to interventional procedures, including permission for the use of medical data in scientific research. All procedures were conducted in accordance with the ethical standards of the institutional research committee and with the 1964 Declaration of Helsinki and its later amendments.

Patients aged 18 years or older who presented to the Pain Clinic of Ankara University Faculty of Medicine, İbni-Sina Hospital, between 1 January 2017 and 1 January 2021 for the indications mentioned above, were retrospectively reviewed using the hospital’s electronic medical record system and the pain follow-up forms maintained in the clinic.

The primary outcome of the study was defined as the change in Numeric Rating Scale (NRS) score from baseline to 12 months after the intervention. Secondary outcomes included changes in NRS scores at 1 and 6 months and changes in McGill Pain Questionnaire (MPQ) scores over the same follow-up period.

Patients were included in the study if they had a Numeric Rating Scale (NRS) pain score of 7 or higher, had completed the McGill Pain Questionnaire (MPQ), had no missing follow-up data, and had undergone a thoracic sympathetic block [1]. Patients under the age of 18, those with chronic inflammatory diseases, or those with a history of thoracic vertebral surgery were excluded. Because only patients with complete 12-month follow-up data and fully documented pain questionnaires were included, selection bias cannot be excluded.

NRS and McGill Pain Questionnaire (MPQ) scores at the time of initial clinic presentation, as well as at the 1st, 6th, and 12th months after the interventional procedure, were evaluated. The Numeric Rating Scale (NRS) is a segmented numeric version of the visual analog scale (VAS), in which patients rate their pain on a scale between 0 and 10 by selecting the whole number that they believe best reflects the intensity of their pain.

The McGill Pain Questionnaire (MPQ) is a multidimensional, self-reported tool used to assess pain in individuals with various diagnoses. It investigates both the intensity and qualitative dimensions of pain. The MPQ comprises three main components: the intensity of sensory descriptors, the cognitive-evaluative aspect of the pain experience, and its affective-emotional impact.

Pain localization and dermatomal distribution were reviewed from medical records. Patients with postherpetic neuralgia and malignancy-related pain reported pain predominantly involving thoracic dermatomes between T2 and T8. Patients with CRPS presented with sympathetically maintained pain affecting the upper extremities, for which a thoracic sympathetic block was deemed clinically appropriate.

**Figure 1:**
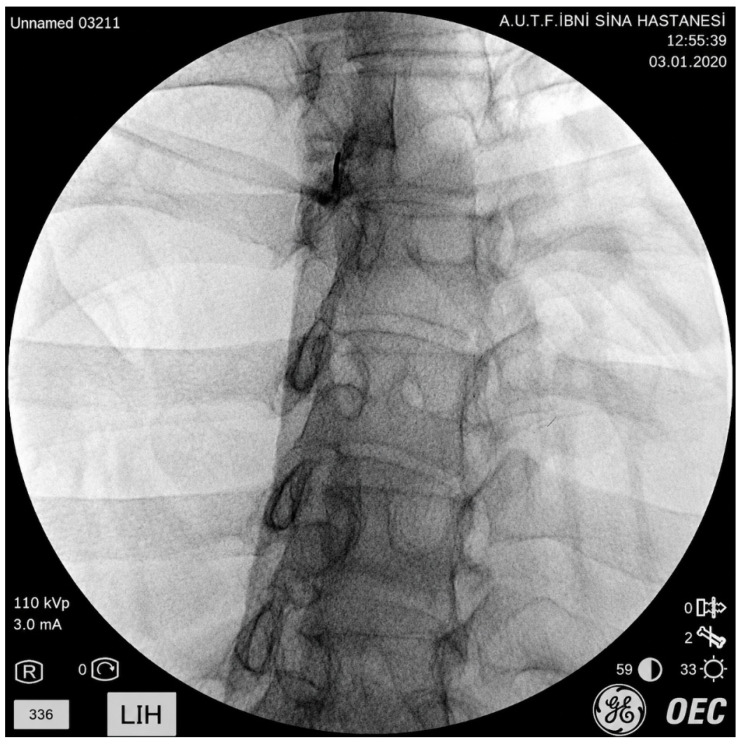
Final needle position and spread of contrast agent.

All procedures were conducted in an operating room under sterile conditions, with local anesthesia and sedation, and fluoroscopic guidance using an intercostal-oblique approach. Following appropriate antiseptic preparation in the prone position, local anesthesia was achieved using 5 mL of 2% lidocaine. With the patient in the anteroposterior position, the T2 vertebra was identified, and the costovertebral junction was targeted as the needle entry site. The needle was advanced to the posterior one-third of the vertebral body. Intravascular or pulmonary spread was ruled out with contrast injection (radio-opaque material should appear as a linear distribution along the vertebra). Once accurate needle placement was confirmed, either a block (2 mL of 0.5% bupivacaine and 8 mg/2 mL dexamethasone) or conventional radiofrequency thermocoagulation (70 °C for 60 s) was performed (Figure 1). Radiofrequency thermocoagulation was applied to patients who responded positively to diagnostic block therapy (at least a 50% reduction in NRS scores) and to patients with malignancy.

## 3. Statistical Analysis

The statistical analysis of the findings was performed using the SPSS 24.0 software (SPSS Inc., Chicago, IL, USA). A significance level of *p* < 0.05 within a 95% confidence interval was considered statistically significant. The normality of data distribution was assessed using the Kolmogorov–Smirnov and Shapiro–Wilk tests. Data were determined to be normally distributed based on *p*-values higher than 0.05 in both tests and skewness and kurtosis values within the acceptable range of −2 to +2. Descriptive statistics (frequency, mean, percentage, minimum, maximum, and standard deviation) were used to evaluate the sociodemographic characteristics of participants. Scale scores are reported as mean, standard deviation, and minimum-maximum values. In addition, to examine the relationship between the pain scores and assessment tools over time, changes in patients’ pain levels were analyzed using the Paired Samples *t*-test.

Given the retrospective nature of the study and the limited sample size, changes in pain scores over time were analyzed using Paired Samples *t*-tests comparing each follow-up with baseline.

## 4. Results

The demographic characteristics of the patients are presented in Table 1. Of the study population, 12 patients (54.5%) were diagnosed with complex regional pain syndrome (CRPS), 4 patients (18.2%) with malignancy (esophageal cancer), and 6 patients (27.3%) with postherpetic neuralgia (Table 1). The mean NRS score before the procedure was 8.59, whereas the scores recorded in the 1st month, 6th month, and 12th month after the procedure were 3.68, 2.50, and 2.05, respectively (Table 2). All changes in NRS scores were found to be significant (*p* < 0.001).

The mean pre-procedure McGill Pain Questionnaire (MPQ) score was found to be 68.05. At the one-month follow-up, the mean MPQ score was 37.27, 24.14 in the 6th month, and 11.09 in the 12th month (Table 3). All changes in MPQ scores were also significant (*p* < 0.001). In patients with refractory pain who responded positively to the diagnostic nerve block and demonstrated at least a 50% reduction in NRS scores (e.g., malignancy-related pain), treatment strategies such as neurolytic block (typically using 4–6 mL of 6–8% phenol solution) or radiofrequency ablation may be preferred.

## 5. Discussion

**Figure 2:**
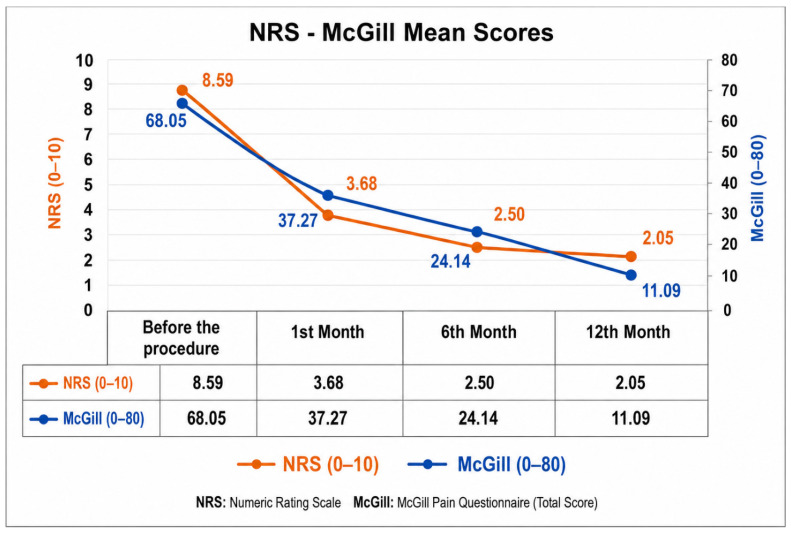
Changes in patients’ pain intensity over time according to the NRS and McGill pain scales.

In this retrospective study, the efficacy of thoracic sympathetic block performed under fluoroscopic guidance was evaluated in patients with chronic pain. The results indicate that the interventional procedure produced a significant reduction in pain intensity (Figure 2). The marked decrease in scores on both the NRS and the MPQ indicates an improvement not only in pain intensity but also in pain quality. These findings are consistent with the literature supporting the effectiveness of thoracic sympathetic block in chronic pain management. The thoracic sympathetic chain continues from the stellate ganglion down to the 12th thoracic vertebra, typically located in the costotransverse region up to T10, and on the anterolateral surface of the vertebral body at T1 and T2 [2].

Leriche first performed a sympathetic block using a paravertebral technique in 1925 [3]. In 1926, Mandl applied the sympathetic block for visceral pain and angina pectoris using the same paravertebral approach. In 1979, Wilkinson revised the thoracic sympathetic block technique and developed radiofrequency thermocoagulation with fewer complications [4]. In the present study, the block was performed via an intercostal-oblique approach. Some centers utilize computed tomography (CT) for this purpose. One of the advantages of CT is the precise and safe needle placement [5].

At our institution, the procedure is routinely performed under fluoroscopic guidance. Advantages of this technique include avoiding intravascular injection, minimizing the risk of pneumothorax due to excessive anterior needle placement, lower radiation exposure compared to CT, shorter procedure duration, and reduced cost [6]. In this study, all procedures were successfully completed under fluoroscopic guidance. No major procedure-related complications were documented in the medical records. However, minor transient adverse events may have been underreported due to the retrospective nature of the study. Even though a small volume of local anesthetic is generally sufficient in thoracic sympathetic blocks, studies have shown that adding steroids to the local anesthetic may improve long-term pain control [7,8]. Bupivacaine is the preferred agent thanks to its longer duration of action [9]. In this study, a mixture of 2 mL of 0.5% bupivacaine and 8 mg dexamethasone was administered for a thoracic sympathetic block.

Thoracic sympathetic block or radiofrequency thermocoagulation is a commonly utilized technique for various chronic pain conditions. These include complex regional pain syndrome (CRPS), postamputation pain syndrome, neuropathic pain syndromes, postherpetic neuralgia, and cancer-related pain, particularly pain associated with esophageal cancer (T2–T8) which is a clear indication for thoracic sympathetic block [10,11,12,13]. Given the results achieved in this study, 12 patients were diagnosed with CRPS, 4 with malignancy (esophageal cancer), and 6 with postherpetic neuralgia. Previous studies supported the use of thoracic sympathetic block as an effective therapeutic option particularly in cases of CRPS, postherpetic neuralgia, and malignancy-related pain [14,15]. Similarly, in the present study, significant reductions in NRS scores were observed in patients with CRPS (54.5%) and in those with other etiologies. The success of sympathetic blocks in managing pain, particularly in neuropathic conditions like CRPS where the pain is often sympathetically maintained, were frequently emphasized in the literature [16].

Furthermore, thoracic sympathetic block was reported to contribute to pain control in malignancy-associated pain, potentially reducing the need for systemic analgesics [17]. The favorable outcomes observed in the patients involved in the present study and having esophageal cancer suggest that this approach may also be beneficial in cancer-related pain management.

The use of both the Numeric Rating Scale (NRS) and the McGill Pain Questionnaire (MPQ) in this study enabled a multidimensional assessment of pain, not only in terms of its intensity but also its qualitative features and the emotional perception of patients. As a multidimensional instrument, the MPQ evaluates the sensory, cognitive, and affective components of pain, thus allowing the monitoring of both quantitative and qualitative changes in pain experience [1]. In this context, the observed improvements in both the overall intensity of pain and its emotional impact represent comprehensive indicators of treatment efficacy. The originality of this study lies in the presentation of 12-month real-world outcomes from a tertiary pain center using both unidimensional and multidimensional pain assessment tools, allowing a comprehensive evaluation of long-term treatment effectiveness.

In patients who are refractory to treatment and who benefit from diagnostic blocks, especially those with malignancy-related pain, the efficacy of neurolytic agents (such as phenol) and radiofrequency ablation is well-documented in the literature [18,19,20]. Similarly, the present study highlights the feasibility and effectiveness of these interventions, suggesting the need to diversify treatment strategies.

Long-term pain control achieved through neurolytic block and radiofrequency ablation not only improves patients’ quality of life but also reduces the need for analgesic medications [21]. However, the potential risks of complications and neurological adverse effects associated with these procedures must be considered. Therefore, careful patient selection and meticulous procedural technique are of paramount importance.

The study included patients with CRPS, postherpetic neuralgia, and malignancy-related pain, conditions with differing pathophysiological mechanisms and expected responses to sympathetic blockade. Due to the limited sample size and retrospective design, formal subgroup analyses according to diagnosis were not feasible. Therefore, results are presented as pooled outcomes and should be interpreted as exploratory.

The retrospective design of this study imposes certain limitations on the interpretation of the results. The absence of a control group and the possibility of data loss inherent to retrospective data analysis may restrict the generalizability of the findings. Nevertheless, the combined use of objective and subjective pain assessment tools enhances the reliability of the results. The detailed demographic data on participants’ age, sex, and diagnostic distribution provide transparency in the sample structure and facilitate comparison with similar populations. Still, the relatively small sample size (*n* = 22) may limit the statistical power of the study. Confirmatory findings from prospective and controlled studies are necessary to further optimize treatment protocols. Moreover, systematic monitoring of long-term efficacy and potential adverse effects is recommended.

We acknowledge that Paired Samples *t*-tests do not fully account for within-subject correlations across multiple time points and may increase the risk of type I error. Future prospective studies with larger samples should employ repeated-measures or mixed-effects models to more robustly analyze longitudinal pain outcomes.

Due to the retrospective design, detailed and standardized data regarding concomitant pharmacological treatments, physical therapy, or additional interventional procedures during follow-up were not consistently available. These co-interventions may have influenced pain trajectories and represent an important limitation of the study.

The results achieved in this study demonstrate that thoracic sympathetic blockade is an effective method for managing chronic pain, yielding significant improvements in pain control. As a component of a multimodal pain management approach, sympathetic blocks are particularly advocated in cases that do not respond to conservative treatments. Furthermore, the demonstrated effectiveness of neurolytic blocks and radiofrequency ablation following diagnostic blockade offers expanded treatment options and facilitates individualized therapeutic planning. These interventions not only alleviate pain intensity but also enhance patients’ functional status.

Several methodological limitations should be considered when interpreting the present findings. First, the retrospective design limits control over data collection and introduces the possibility of missing or incompletely documented variables. Second, the relatively small sample size and absence of a control group reduce statistical power and limit the generalizability of the results. Third, the study population included patients with heterogeneous pain etiologies, including CRPS, postherpetic neuralgia, and malignancy-related pain, which may differ in their underlying mechanisms and responsiveness to sympathetic blockade. In addition, both diagnostic block and radiofrequency thermocoagulation were performed according to routine clinical practice, resulting in procedural heterogeneity. Detailed and standardized information regarding concomitant pharmacological treatments, physical therapy, and additional interventional procedures during follow-up was not consistently available, and these co-interventions may have influenced the observed pain outcomes. Furthermore, because only patients with complete 12-month follow-up data were included, selection bias cannot be excluded. Therefore, the observed reductions in pain scores should be interpreted as exploratory findings that require confirmation in larger prospective controlled studies.

## 6. Conclusions

Future studies should examine the efficacy and safety of thoracic sympathetic blockade through larger, controlled, and prospective research designs. Investigating differential responses based on pain types (e.g., neuropathic vs. inflammatory) could support the development of more personalized treatment protocols. Comprehensive data on the long-term outcomes and complication profiles of radiofrequency ablation and neurolytic block techniques are also necessary. Assessing their effect on patients’ quality of life and functional outcomes will provide valuable support for clinical decision-making.

Finally, advancing molecular and neurophysiological research into the mechanisms of sympathetic blocks will contribute significantly to defining more precise therapeutic targets.

## Figures and Tables

**Table 1:** Demographic characteristics of the participants.

		*n*	%
Age (X ± SD (min-max)		50.13 ± 14.27 (min 31-max 74)	
Sex	Female	15	68.2
	Male	7	31.8
	Total	22	100
Height (X ± SD (min-max)) (cm)		169.27 ± 4.74 (min 160-max 178)	
Weight (X ± SD (min-max)) (kg)		72.04 ± 7.68 (min 55-max 85)	
BMI (X ± SD (min-max))		25.13 ± 2.43 (min 19-max 29.40)	
Diagnosis	Postherpetic Neuralgia CrpsEsophagus Cancer Total	6	27.3
		12	54.5
		4	18.2
		22	100

Values are presented as mean ± SD, number (%); BMI = body mass index; SD: standard deviation; CRPS: complex regional pain syndromes.

**Table 2:** Patients’ NRS scores at preoperative, 1st-month, 6th-month, and 12th-month evaluations.

Time	NRS X ± SD (Min-Max)	*p*
Preop	8.59 ± 1.14 (min 7, max 10)	*p* < 0.001
1st month	3.68 ± 0.99 (min 2, max 5)	*p* < 0.001
6th month	2.50 ± 0.51 (min 2, max 3)	*p* < 0.001
12th month	2.05 ± 0.72 (min 1, max 3)	*p* < 0.001

Values are presented as mean ± SD, number (%); SD = standard deviation; Paired Samples *t*-test.

**Table 3:** Patients’ McGill Pain Questionnaire scores at preoperative, 1st-month, 6th-month, and 12th-month evaluations.

Time	McGill X ± SD (Min-Max)	*p*
Preop	68.05 ± 5.05 (min 56, max 76)	*p* < 0.001
1st month	37.27 ± 4.46 (min 27, max 45)	*p* < 0.001
6th month	24.14 ± 3.21 (min 18, max 30)	*p* < 0.001
12th month	11.09 ± 2.32 (min 8, max 17)	*p* < 0.001

Values are presented as mean ± SD; SD: standard deviation; Paired Samples *t*-test.

## Data Availability

All data generated or analyzed during this study are included in this published article.

## References

[1] Melzack R. (1975). The McGill Pain Questionnaire: Major properties and scoring methods. Pain.

[2] Raj P.P., Lou L., Erdine S., Staats P.S., Lampert R. (2003). T2 and T3 sympathetic nerve block and neurolysis. Radiographic Imaging for Regional Anesthesia and Pain Management.

[3] Leriche R., Fontaine R. (1934). L’anesthesie isolee du ganglion etoile: Sa technique, ses indications, ses resultats. Presse Méd..

[4] Wilkinson H.A. (1996). Percutaneous radiofrequency upper thoracic sympathectomy. Neurosurgery.

[5] Guo J.G., Fei Y., Huang B., Yao M. (2016). CT-guided thoracic sympathetic blockade for palmar hyperhidrosis: Immediate results and postoperative quality of life. J. Clin. Neurosci..

[6] Koizuka S., Nakajima K., Mieda R. (2014). CT-guided nerve block: A review of the features of CT fluoroscopic guidance for nerve blocks. J. Anesth..

[7] Stanton-Hicks M. (2001). Thoracic sympathetic block: A new approach. Tech. Reg. Anesth. Pain Manag..

[8] Doroshenko M., Turkot O., Dua A., Horn D.B. (2025). Sympathetic Nerve Block. StatPearls.

[9] Yektaş A. (2017). Bupivakain-lidokain karışımının neden olduğu sistemik lokal anestezik toksisitesinde İntravenöz lipid ile tedavi. Okmeydanı Tıp Derg..

[10] Raj P.P., Lou L., Erdine S., Staats P.S., Lampert R. (2003). Celiac plexus block and neurolysis. Radiographic Imaging for Regional Anesthesia and Pain Management.

[11] Raj P.P., Lou L., Erdine S., Staats P.S., Lampert R. (2003). Lumbar sympathetic block and neurolysis. Radiographic Imaging for Regional Anesthesia and Pain Management.

[12] Raj P.P., Lou L., Erdine S., Staats P.S., Lampert R. (2003). Hypogastric plexus block and neurolysis. Radiographic Imaging for Regional Anesthesia and Pain Management.

[13] Raj P.P., Lou L., Erdine S., Staats P.S. (2003). Ganglion impar block. Radiographic Imaging for Regional Anesthesia and Pain Management.

[14] de Oliveira Rocha R., Teixeira M.J., Yeng L.T., Cantara M.G., Faria V.G., Liggieri V., Loduca A., Müller B.N., Souza A.C.M.S., de Andrade D.C. (2014). Thoracic sympathetic block for the treatment of complex regional pain syndrome type I: A double-blind randomized controlled study. Pain.

[15] Kumar V., Krone K., Mathieu A. (2004). Neuraxial and sympathetic blocks in herpes zoster and postherpetic neuralgia: An appraisal of current evidence. Reg. Anesth. Pain Med..

[16] Birklein F. (2005). Complex regional pain syndrome. J. Neurol..

[17] Mercadante S., Klepstad P., Kurita G.P., Sjøgren P., Giarratano A. (2015). Sympathetic blocks for visceral cancer pain management: A systematic review and EAPC recommendations. Crit. Rev. Oncol. Hematol..

[18] Fumić Dunkić L., Hostić V., Kustura A. (2022). Palliative Treatment of Intractable Cancer Pain. Acta Clin. Croat..

[19] Uchida K. (2009). Radiofrequency treatment of the thoracic paravertebral nerve combined with glucocorticoid for refractory neuropathic pain following breast cancer surgery. Pain Physician.

[20] Gulati A., Shah R., Puttanniah V., Hung J.C., Malhotra V. (2015). A retrospective review and treatment paradigm of interventional therapies for patients suffering from intractable thoracic chest wall pain in the oncologic population. Pain Med..

[21] Filippiadis D.K., Tselikas L., Bazzocchi A., Efthymiou E., Kelekis A., Yevich S. (2020). Percutaneous Management of Cancer Pain. Curr. Oncol. Rep..

